# Supplementary material for: Cervical length distribution among Brazilian pregnant population and risk factors for short cervix: A multicenter cross-sectional study
Source: PLoS One. 2022 Oct 7;17(10):e0272128. doi: 10.1371/journal.pone.0272128 (PMC9544154; doi:10.1371/journal.pone.0272128)
Supplement: S1 Appendix — (DOCX) [file pone.0272128.s001.docx]

**S1 Appendix. P5 trial description**

The P5 Trial -Pessary Plus Progesterone to Prevent Preterm Birth Study, is a pragmatic, multicentre, open-label randomized controlled trial (Trial registration RBR-3t8prz), testing the effectiveness of pessary plus progesterone versus progesterone alone in reducing preterm birth and a composite of neonatal adverse events. This study was financed by Bill & Melinda Gates Foundation and National Council for Scientific and Technological Development (CNPq) Ministry of Health and approved by Brazilian National Review Board (CONEP - registration 1.055.555). The research assistant, previously trained, collected informed consent before submitting the participant to the ultrasound scan, and in case of the short cervix, another consent was applied before the randomization.

It included singleton and twin pregnancies with gestational age between 18 to 22 weeks and 6 days and no maternal age restriction. Then, it was offered cervical length measurement by ultrasound. Pregnant women with a cervical length below or equal to 30 mm (but more than 5 mm) are eligible for the trial. Exclusion criteria are painful contractions, vaginal bleeding, cerclage during current pregnancy before the screening, preterm premature rupture of membranes, severe liver disease, cholestasis during this pregnancy, previous or current thromboembolism, placenta previa, cervical dilation greater than 1 cm, monoamniotic twin pregnancy, higher order twin pregnancy (triplets or higher), major fetal malformation of at least one fetus and stillbirth.

All participating members of the research team were trained to perform the cervical measurement by ultrasound (Fetal Medicine Foundation training program) and to manage the pessary. Women with a short cervix and without any exclusion criteria were invited to participate in the randomized clinical trial. The pregnant women were randomized into two groups: pessary plus progesterone or progesterone alone.

This study is in the results evaluation phase.
