# Supplementary material for: Cervical length distribution among Brazilian pregnant population and risk factors for short cervix: A multicenter cross-sectional study
Source: PLoS One. 2022 Oct 7;17(10):e0272128. doi: 10.1371/journal.pone.0272128 (PMC9544154; doi:10.1371/journal.pone.0272128)
Supplement: S3 Appendix — (DOCX) [file pone.0272128.s003.docx]

**S3 Appendix. P5 working group**

Amanda Dantas; Anderson Borovac-Pinheiro; Antonio Fernandes Moron; Carlos Augusto Santos Menezes; Cláudio Sérgio Medeiros Paiva; Cristhiane B Marques; Cynara Maria Pereira; Djacyr Magna Cabral Paiva; Elaine Christine Dantas Moisés; Enoch Quinderé Sá Barreto; Felipe Soares; Fernando Maia Peixoto-Filho; Francisco Edson de Lucena Feitosa; Francisco Herlanio Costa Carvalho; Jessica Scremin Boechem; João Renato Benini-Jr.; Karayna Gil Fernandes; Kleber Cursino Andrade; Leila Katz; Maíra Rossmann Machado; Marcelo L Nomura; Marcelo Marques Souza Lima; Marcelo Santucci Franca; Marcos Nakamura-Pereira; Maria Julia Miele; Maria Laura Costa; Mário Dias Correia Jr; Nelson Sass; Renato T Souza; Rodrigo Pauperio Soares Camargo; Samira Maerrawi Haddad; Sérgio Martins-Costa; Silvana F Bento; Silvana Maria Quintana; Stéphanno Gomes Pereira Sarmento;
