# Supplementary material for: Cervical length distribution among Brazilian pregnant population and risk factors for short cervix: A multicenter cross-sectional study
Source: PLoS One. 2022 Oct 7;17(10):e0272128. doi: 10.1371/journal.pone.0272128 (PMC9544154; doi:10.1371/journal.pone.0272128)
Supplement: Attachment — Submitted filename: Response to Reviewers_rcp.docx [file pone.0272128.s005.docx]

Campinas, November 17, 2021

Dear Dr. Federico Ferrari
Academic Editor
PLOS ONE,

Thank you for the revision of our article. We appreciate all suggestions, and we are sure that these have contributed for the quality of our study, making it clearer and completing information that were lacking on the last version.

We have read your comments and the comments of the reviewers with interest and tried to adjust the manuscript accordingly. Included you will find two revised drafts of the manuscript. In one of these versions the adjustments have been highlighted in the revised manuscript. A point-by-point response to reviewers follows.

All authors have read and approved the revised version of the manuscript.

We hope that our adjustments make the manuscript suitable for publication in the journal, and we are looking forward to your final decision on the manuscript.

Please contact us for further clarification in any doubts.

Yours sincerely,

On behalf of the authors

Rodolfo Pacagnella, MD, Ph.D.

Point-by-point responses

Reviewer 1

Abstract
A brief mention of why this topic is important would be helpful.
Thank you for the suggestion. We believe that the importance of this study is in contributing to screening strategies since there are populational differences and risk factors that influence the cervical length (line 69-72). We considered this

The mention of straight versus curved measurements does not appear to align with the rest of the abstract. This should be clearer.

As for the alignment of the straight and curved technique, we made changes to the text to make it clear that for statistical analysis (logistic regression), we used the straight-line technique, since it is the gold standard technique for performing measurements of the uterine cervix. We used the curved technique only to compare with the standard technique. We excluded this information from the abstract as this could bring confusion.

Intro
Page 5 - Second paragraph - recommend mentioning ACOG's use of 25 mm cutoff

Thank you very much. We acknowledge that ACOG’s uses 25 mm as the cutoff point for a short cervix and we included this reference: Prediction and Prevention of Spontaneous Preterm Birth: ACOG Practice Bulletin, Number 234. Obstet Gynecol. 2021;138(2):e65-e90.

Page 5 - Paragraph 3 is well written but I would also include mention of the techniques for measuring CL. What does AIUM say? How did previous publications describe their approach to CL measurements?

Thank you for the comment. We added a mention about the technique in the first paragraph to make it clearer that transvaginal ultrasound is the gold standard for measuring the cervix and that the straight-line technique is the established method.

The AIUM also advises that transvaginal ultrasound is the best method for evaluating the cervix and describes the technique for measuring the cervix in straight line. Thus, we add the following reference: AIUM Practice Parameter for the Performance of Limited Obstetric Ultrasound Examinations by Advanced Clinical Providers. J Ultrasound Med. 2018;37(7):1587-96.

As already established, the straight technique is the standard measure [2-4, 10, 15-17]. However, previous studies [18, 23] have already shown that the straight measurement can underestimate the measurement of the cervix, since longer cervix tend to adopt a more curvilinear shape. The curved measurement, as it follows the anatomy of the cervix, gives us a real measure of the cervical length. Our study confirmed these findings. Our objective was to construct distribution curves for the uterine cervix of Brazilian pregnant women in the second trimester, and a multicenter study had never been carried out in Brazil. Therefore, we think it would be important to compare the technique that respects the anatomy (curved technique) with the standard technique. However, as the short cevix tends to assume a straightened anatomical shape, we observe that the straight technique remains an effective method of diagnosis.

Page 6 - First paragraph would use "distribution curves" or "population curves" as the wording is confusing

We changed to distribution

Materials/Methods
I believe this is a case-control study

We understand the study design as a cross-sectional study. Although we identified short and no-short cervices within our population, we obtained data from all the participants (cervical measurement, sociodemographic data, and obstetric and gynecological history) regardless any characteristics of cervical length. We did not select our study population considering cervical length, we performed the group identification in the analysis only.

Why were so many types of subjects excluded?

Thank you for your question. We used data from a large randomized, multicenter study that aimed to compare the effectiveness of using progesterone versus progesterone and pessary in patients with a short cervix. Therefore, as our study is a secondary analysis of this large study, it was necessary to follow the exclusion criteria of the main study and, from this database, we only excluded twin pregnancies.

Page 7 Paragraph 3 - what is the reference for the training?

We appreciate your suggestion. We added the reference: The Fetal Medicine Foudation. The FMF certification cervical assessment. Available in: <https://fetalmedicine.org/fmf-certification-2/cervical-assessment-1>. Accessed on November 2, 2021.

Results
Page 12 - The mention of cervical volume was not described in the intro and its utility/relevance was not described

Thank you very much. We added one paragraph in the introduction: “The standard technique for measuring the cervix using TVU is to draw a straight line between the internal and external os [1-4]. Previous studies have already compared the straight technique with the curved technique [23], as well as the contribution of the volume of the uterine cervix for the diagnosis of short cervix. No technique showed better results compared to the standard technique [24-29]”.

We also described in the discussion that volume of the cervix was not relevant for diagnosis of short cervix, and we observed that short cervix tend to be rectified so curve straight line could be the best strategy.

Was an adjusted analysis done to assess for confounders? Were Tables 3/4/5 adjusted?

Yes, an adjusted analysis was performed to assess confounding factors. Tables 3 and 4 are analyzes of pre-determined subgroups in the study design and worked statistically with multiple logistic regression to reduce confounding factors.

Table 5 isn't labeled in the text

There was no table 5, however, I agree that the way table 4 was structured was confusing. In this way, we broke down table 4 and created table 5. Thanks for the suggestion.

Data points should be shown as bell curves as well and authors should consider overlapping these images

We understand that presenting data as we present in the text may be more useful for clinicians to use the curves as presenting in a bell shape.

Some of the results are confusing regarding the difference in the two measurement techniques.

We tried to reduce the confusion changing some wording and the data in tables.

Discussion
Which values did the authors use the straight line or the curved for the discussion?

Thank you very much for your observation, it was not clearly written. We used the straight technique for the analysis, which is the standardized technique for measuring the cervical length of the uterine cervix. We added this information in the first paragraph to make it clearer: “We determined the CL distribution (in straight line) among second trimester Brazilian pregnant women”.

Comparison of previous studies evaluating cervical volume to their results would benefit this manuscript.

We compared with other studies that also evaluated volume of the cervix, however, it has not been shown to be superior to diagnose short cervix compared to the standard method (straight technique): “We also observed that the volume of the cervix increased slightly over the course of gestation despite the progressive shortening of the longitudinal measurement of the cervix. In other words, the cervix becomes shorter but wider [23]. Although many studies have shown a correlation between cervical volume and the ability of this measure to contribute to the prediction of the risk of prematurity, none has demonstrated additional benefits in relation to the longitudinal cervical measurement technique [24-28].”

Comparison of previous studies evaluating techniques for cervical length measurement would benefit this manuscript

Thank you for your suggestion. We agreed and added comparison of previous study: “Similar results were found in a prospective Dutch cohort involving 508 women aged 18-22 weeks who identified greater differences between line and trace measurement techniques above P95 (line 51.1mm x trace 55mm, p <0.0001) [21]. This result is because the straight measurement underestimates the biometry of the cervix. The construction of the reference curve for the measurement of the cervix, considering the straight line as a reference, specially underestimates the values above 75^th^ percentile when the difference between the straight and curve measurements assumes a tendency to be > 4mm, which according to the Fetal Medicine Foundation (FMF) protocol cannot be considered an operator-dependent difference [15].”

Majority of discussion and conclusion are well written

Thank you very much.

Reviewer 2

Line 45-47: I don’t understand what are they saying, please work on it and more informative.

Thank you for your suggestion. We tried to rewrite more clearly.

" Since there are populational differences and risk factors that influence the cervical length, the aim of the study was to construct a populational curve with measurements of the uterine cervix of pregnant women in the second trimester of pregnancy and to evaluate which variables was related to cervical length ≤25 mm".

Line 54-55: Didn’t show in paper those are not significant to your research.

Thank you very much for your observation.

"showed similar results: range 21.0–25.0 mm in straight versus 22.6– 26.0 mm in curve measurement for the 5th percentile".

Line 76: Review these references to fit the journal guidelines and also Please check grammar.

We adapted the references according to the journal guidelines.

"TVU can also help to prevent preterm birth (PTB) because cervical length is one of the best predictors of preterm birth, and short cervical length may trigger interventions"

Line 78: Cannot use this type of word. Please review it.

Thank you. We rephrased the sentence.

"The progesterone has a role in reducing spontaneous preterm in singleton pregnancies with cervical length (CL) ≤ 25 mm".

Line 109-186: In material and method section is not clear, rewrite again kindly, for data analysis which software are authors use?

We rephrased some sentences in order to make It clear. For data analysis we used the software “R” form R Foundation for Statistical Computing.

Line 159-167: Don’t use figure in methods and where is your statical analysis? and check grammar.

Thank you for your guidance. This study used ultrasonography to obtain measurements of the cervix, and in addition there are different techniques to measure the cervix. Therefore, we believe this is a clear way of exemplifying how we obtained the measurements. Other references used figure in methods:

Heath VC, Southall TR, Souka AP, Novakov A, Nicolaides KH. Cervical length at 23 weeks of gestation: relation to demographic characteristics and previous obstetric history. Ultrasound Obstet Gynecol. 1998;12(5):304-11.

The last two paragraphs of material and methods section describe statistical analyzes

 Line 189-191: Please add these lines in “material and method” section

I appreciate your comment. In this study, we used the STROBE checklist (STrengthening the Reporting of Observational studies in Epidemiology). In the results, item number 13, the description of the participants is oriented: (a) Report numbers of individuals at each stage of study—eg numbers potentially eligible, examined for eligibility, confirmed eligible, included in the study, completing follow-up, and analyzed.

Line 192: Don’t use figure, please rewrite this.

The STROBE suggests considering the possibility of use a flow diagram. We chose to use the flowchart to better illustrate eligible and excluded participants.

 Line 227-238: Didn’t need use any figure here.

I really consider your comments, however, as the main objective of this study was to construct curves with measurements of the cervix of pregnant women in the second trimester of pregnancy, we thought it would be interesting to present such curves.

Line 245: Check Grammar

Thank you for your comment. checked.

Line 277: Check Grammar

Thank you.

checked.

 Line 296: Check the spelling

We corrected the spelling: “shorter cervix”.

Line 327 – 332: The first sentence is not clear. It seems incomplete. Rewrite the full lines.

We rewrote the sentence:

“We found that CL was shorter in pregnant women ≤19 years old than those >20 years old. In the literature, young pregnant women are at increased risk for spontaneous PTB [30], which may be due to biological immaturity of the female genital tract [31,32], social and behavioral factors [18], and intra-amniotic infections as a consequence of genital tract infections [31]”.

Line 378-383: Rewrite the conclusion again

Thank you for your suggestion. We rewrote the conclusion.

“The reference CL distribution curves should consider populational characteristics since physicians may use it as a strategy to prevent preterm birth in clinical practice. Doing so, it will enable a more efficient diagnosis of short cervix and its association with prematurity, allowing assertive medical decisions. Moreover, we suggest that subsequent studies should consider these populational characteristics to build new distribution curves and define specific screening strategies for different populations to prevent premature delivery”.
