# Supplementary material for: Cervical length distribution among Brazilian pregnant population and risk factors for short cervix: A multicenter cross-sectional study
Source: PLoS One. 2022 Oct 7;17(10):e0272128. doi: 10.1371/journal.pone.0272128 (PMC9544154; doi:10.1371/journal.pone.0272128)
Supplement: Attachment — Submitted filename: Review Comments_Revised.docx [file pone.0272128.s006.docx]

In this manuscript, Marquat and collaborators present results of the association between cervical length distribution and risk factors for short cervix. The study was conducted with 8,167 pregnant women in Brazil. The authors should correct some simple errors before publication.

Line 47: Have spelling in this word “secund”, it will be ‘second’.

Line 90: Use just one space before ‘including’ word; It will be ‘American College of Obstetrics and Gynecology (ACOG)’.

Line 195: Please rewrite this sentence as like “Statistical analysis is performed using R software from the R Project for Statistical Computing (version *.*.0)”.

Line 254: Please check grammar.

Line 286: Please check grammar.

Line 343: Please rewrite “straight-line”.

Line 363: Use just one space.

Line 365: Use just one space.

Line 414: Rewrite “birth in”.
