# Supplementary material for: Cervical length distribution among Brazilian pregnant population and risk factors for short cervix: A multicenter cross-sectional study
Source: PLoS One. 2022 Oct 7;17(10):e0272128. doi: 10.1371/journal.pone.0272128 (PMC9544154; doi:10.1371/journal.pone.0272128)
Supplement: Attachment — Submitted filename: Response to Reviewers_kgfm.docx [file pone.0272128.s007.docx]

Campinas, June 2, 2022

Emily Chenette

Editor-in-Chief

Plos ONE

Dear Dr.

Thank you for the revision of our article. We appreciate the suggestions, and we are sure that these have contributed to the quality of our study, making it clearer and completing information that was lacking in the last version.

We have read your comments and the comments of the reviewers with interest and tried to adjust the manuscript accordingly. Included you will find two revised drafts of the manuscript. In one of these versions, the adjustments have been highlighted in the revised manuscript. A point-by-point response to reviewers follows.

All authors have read and approved the revised version of the manuscript.

We hope that our adjustments make the manuscript suitable for publication in the journal, and we are looking forward to your final decision on the manuscript.

Please contact us for further clarification in any doubts.

Yours sincerely,

On behalf of the authors

Rodolfo Pacagnella, MD, Ph.D.

Point-by-point responses

Reviewer 1

Intro
Line 82 - word "birth" is missing after preterm

**R:** Thank you for the suggestion. We rewrote: “TVU can also help to prevent prematurity because cervical length is one of the best predictors of preterm birth (PTB)”

Line 90 - "Obstetricians and Gynecologists"

**R:** Checked.

Methods
What is the power calculation for exactly? rate of CLs <25 mm? preterm birth?

**R:** The objective is to know the profile of measurements of the cervical length of Brazilian pregnant women in the second trimester of pregnancy and, consequently, which variables are related to measurements of the cervix ≤ 25 mm. We know that cervical measurements ≤ 25 mm are related to an increased risk for preterm birth.

What was the primary outcome?

**R:** The primary outcome is the construction of a distribution curve for cervical length measurements of Brazilian pregnant women in the second trimester of pregnancy (between 18 to 22 weeks and 6 days) using ultrasound (straight technique).

I would clarify that there are two separate analyses being described. the two groups are separated by CL <25 and then the use of the different CL measurements.
This section needs to be clearer exactly what was done.

**R:** We appreciate your suggestion. However, there are no two separate groups. We have a population of 7844 pregnant women in the second trimester of pregnancy. Straight-line cervical length measurements were obtained in all of them (standard measurement) and from then on, the percentage of short cervix per variable (maternal age, schooling, marital status, region, race, BMI, obstetric and gynecological history, and gestational age...) was observed. In a secondary analysis, we compared the standard technique with cervical length using the curved technique and the volume of the cervix.

We rewrote it to make it cleare**r:**

“Four strategies of uterine cervical measurements were used in our study: straight-line measurement (SL) between the internal to the external os, used for the primary outcome (distribution); curved measurement (CM) with two straight measurements respecting the endocervical canal pathway between the internal and external os (Fig 1 and 2); anteroposterior measurement near the insertion of the uterine arteries, in the middle third of the cervix; and transverse measurement rotating the transducer 90 degrees to allow transverse visualization of the cervix. The volume of the cervix was calculated using the formula for the volume of a cylinder, *πR²h*, where R is half the transverse diameter of the cervix, and h is the length. The curved measurement and the measurements for calculating the volume were used only for comparison purposes with the standard straight measurement.”

Results
Overall this section is confusing. I think getting rid of some of the text and referring more to tables might help. I think stressing the important findings and leaving the details to the tables would help streamline this section as well. It is hard to identify what the focus of the paper truly is.

**R:** Thank you for the suggestion. We reduced some of the information repeated in the tables.

Line 222 - not sure what this means

**R:** As described in other studies and initially noted by Iams et al., pregnant women with a cervical length ≤25 mm are at increased risk of preterm birth than those with a cervical length > 25 mm. As we calculated the percentile, we are reporting our result that in our population, women with a percentile below the 5th have a cervix ≤25 mm and, consequently, a higher risk of preterm birth.

Fig 4 - why no means or percentages of those <25 mm in the last column for curved measurements?

**R:** Thank you for highlighting this. We completed the missing values in table 2

Tables 3/4/5 - Should these say "multivariable analysis"? if yes, then the text should be changed from "OR" to "aOR"

**R:** Indeed. We changed in the text.

Discussion
Line 291 - Not sure what point they are making regarding the 25th %ile. Is this a higher risk group in your cohort? How is 32mm different from 25th %ile previously described?

**R:** When we compared the lengths of the uterine cervix for the 5th, 10^th^, and 25th percentiles, respectively, we observed values similar to the Iams study. Some studies consider that pregnant women with a uterine cervix measurement <30 mm are at increased risk for preterm delivery, and this value corresponds to the 25th percentile of the Iams study. In our population, pregnant women in the 25th percentile correspond to 32 mm, and therefore, considering the 25th percentile as an increased risk for premature birth, patients with measurement < 32 mm would be at increased risk. We understand that this information may be causing confusion and as such we have chosen to remove it.

Line 302 - How were they different? Why might that be (based on population studied, inclusion/exclusion criteria, etc)?

**R:** We changed the paragraph to adapt it to the suggestion.

The discussion regarding straight vs curved could be shortened and combined.

**R:** We reduced the discussion to fit in two short paragraphs containing important information regarding the issue.

Line 362 - is there data linking this to preterm birth?
Limitations should include exclusion of those with cerclage and who are dilated as this eliminates a large chunk of short cervix patients (this may also explain why your CL %iles might be slightly different in your population if other studies included those subjects)

**R:** Patients under cervical stitches are rare in Brazil and we understand that this would not have influenced int the distribution of cervical length.

Reviewer 2

Line 47: Have spelling in this word “secund”, it will be ‘second’.

**R:** Thank you. We correct.

Line 90: Use just one space before ‘including’ word; It will be ‘American College of Obstetrics and Gynecology (ACOG)’

**R:** Thank you very much. We correct.

Line 195: Please rewrite this sentence as like “Statistical analysis is performed using R software from the R Project for Statistical Computing (version *.*.0)”.

**R:** We rewrote the sentence.

Line 254: Please check grammar.

**R:** Thank you. I believe you are referring to the "were" in this sentence: The variables and percentage of CL ≤25 mm *were* as follows”. However, as the context is in the plural (variables and percentage), that's why "were" is being used instead of "was".

Line 286: Please check grammar

**R:** Checked. We use “were” for the plural (the risk factors were). “Risk factors associated with increased risk of CL ≤25 mm were as follows”

Line 343: Please rewrite “straight-line”.

**R:** Thank you. We rewrote.

 Line 363: Use just one space

**R:** Checked.

Line 365: Use just one space

**R:** Checked.

 Line 414: Rewrite “birth in”

**R:** Thank you. We rewrote.
